# Supplementary material for: GraphOmics: an interactive platform to explore and integrate multi-omics data
Source: BMC Bioinformatics. 2021 Dec 18;22:603. doi: 10.1186/s12859-021-04500-1 (PMC8684259; doi:10.1186/s12859-021-04500-1)
Supplement: Supplementary file 1 — Additional file 1. Supplementary data S1–S6. [file 12859_2021_4500_MOESM1_ESM.pdf]

GraphOmics: an Interactive Platform to Explore and Integrate Multi-omics Data

Joe Wandy[a] and Rónán Daly[a,*]

[a]Glasgow Polyomics, University of Glasgow, Glasgow, G61 1BD, United Kingdom

June 23, 2021

Supplementary Section S1: Input file format

To use GraphOmics, for each omics of interest, users have to prove its measurement data in Comma-separated Values (CSV) format. This takes the form of a matrix, where the first column contains the ID and further columns represent individual samples. For example, the intensity matrix takes the form of:

```
Identifier,sample1,sample2,sample3,sample4,FC_case1_vs_control,padj_case1_vs_control
ENSDARG00000000001,510,402,698,783,1.246,0.000284
ENSDARG00000000002,283,129,164,269,-0.7249,1.27e-06
ENSDARG00000000018,545,503,547,387,-0.0401,0.760679007
```

The following identifiers are accepted:

- Transcripts: Ensembl ID
- Proteins: UniProt ID
- Metabolites: KEGG or ChEBI ID

If statistical tests have been performed for the entities, the results can also be included as columns in the matrix. The column header has to be in the following format: 'FC_<xx>_vs_<yy>' for fold change information, and 'padj_<xx>_vs_<yy>' for p-values.

Finally a design matrix has to be specified to assign samples to factors (experimental conditions). This takes the form of another CSV file in this format:

```
sample,group
sample1,case1
sample2,case1
sample3,control
sample4,control
```

Supplementary Figure S2: Clustergrammer Results for the Zebrafish Data

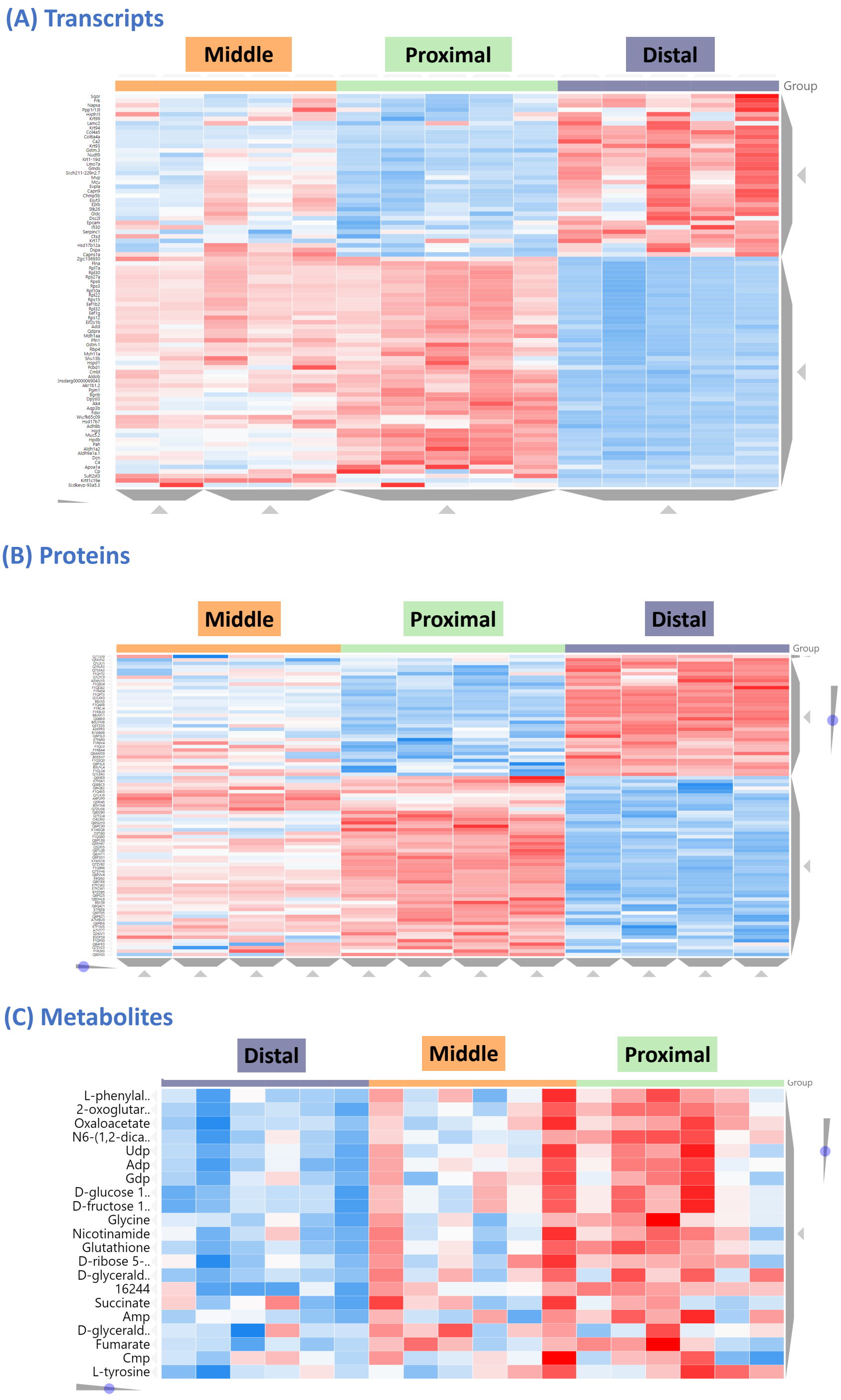

Clustergrammer results for selected transcripts, proteins and metabolites. The Query Builder was used to filter for transcripts and proteins with a threshold of 0.05 on the p-values, and at least ±0.5 on the log fold changes of the transcripts in the distal-vs-proximal comparison. The results were a selection of 87 transcripts and their corresponding proteins, as well as 21 compounds involved in reactions catalysed by those proteins.

Supplementary Table S3: Differentially Expressed Pathways from the Zebrafish Metabolite Data

Reactome ID	Pathways	p-values transcripts	p-values proteins	p-values metabolites
R-DRE-5223345	Miscellaneous transport and binding events	3.11E-03	-	1.91E-03
R-DRE-8964540	Alanine metabolism	9.82E-01	-	2.81E-03
R-DRE-8963684	Tyrosine catabolism	7.46E-03	1.07E-02	3.50E-03
R-DRE-352230	Amino acid transport across the plasma membrane	1.79E-03	-	3.80E-03
R-DRE-420499	Class c/3 (metabotropic glutamate/pheromone recep...	2.10E-02	-	3.93E-03
R-DRE-428559	Proton-coupled neutral amino acid transporters	-	-	4.28E-03
R-DRE-71240	Tryptophan catabolism	1.92E-02	-	4.46E-03
R-DRE-442660	Na+/cl- dependent neurotransmitter transporters	9.74E-04	-	4.59E-03
R-DRE-8964539	Glutamate and glutamine metabolism	3.97E-02	1.00E+00	5.76E-03
R-DRE-70921	Histidine catabolism	5.84E-03	-	5.93E-03
R-DRE-8963693	Aspartate and asparagine metabolism	3.19E-02	1.00E+00	6.58E-03
R-DRE-70895	Branched-chain amino acid catabolism	7.04E-02	1.00E+00	6.80E-03
R-DRE-210455	Astrocytic glutamate-glutamine uptake and metabol...	1.60E-01	-	7.10E-03
R-DRE-446210	Synthesis of udp-n-acetyl-glucosamine	7.19E-01	1.00E+00	7.45E-03
R-DRE-156584	Cytosolic sulfonation of small molecules	7.10E-03	1.00E+00	8.11E-03
R-DRE-561048	Organic anion transport	8.36E-01	-	8.48E-03
R-DRE-5423646	Aflatoxin activation and detoxification	7.59E-03	-	9.32E-03
R-DRE-174403	Glutathione synthesis and recycling	6.94E-03	-	9.82E-03
R-DRE-2142691	Synthesis of leukotrienes (lt) and eoxins (ex)	2.26E-03	-	9.82E-03
R-DRE-5683826	Surfactant metabolism	9.41E-03	3.49E-01	1.04E-02
R-DRE-71288	Creatine metabolism	2.60E-02	-	1.05E-02
R-DRE-549127	Organic cation transport	9.83E-04	-	1.07E-02
R-DRE-196807	Nicotinate metabolism	6.22E-02	-	1.15E-02
R-DRE-196757	Metabolism of folate and pterines	6.81E-02	4.71E-01	1.23E-02
R-DRE-416476	G alpha (q) signalling events	5.72E-04	1.88E-01	1.36E-02
R-DRE-5628897	Tp53 regulates metabolic genes	7.47E-01	9.79E-01	1.36E-02
R-DRE-71064	Lysine catabolism	8.81E-02	1.00E+00	1.41E-02
R-DRE-389661	Glyoxylate metabolism and glycine degradation	9.38E-02	7.85E-01	1.59E-02
R-DRE-70635	Urea cycle	3.48E-01	-	1.61E-02
R-DRE-9639288	Amino acids regulate mtorc1	1.00E+00	1.00E+00	1.68E-02
R-DRE-500753	Pyrimidine biosynthesis	1.92E-01	-	1.72E-02
R-DRE-163754	Insulin effects increased synthesis of xylulose-5...	-	-	1.84E-02
R-DRE-70263	Gluconeogenesis	2.24E-02	8.39E-02	1.85E-02
R-DRE-71336	Pentose phosphate pathway	1.32E-01	4.75E-01	1.86E-02
R-DRE-70350	Fructose catabolism	5.16E-03	-	1.90E-02
R-DRE-196783	Coenzyme a biosynthesis	2.63E-02	-	1.98E-02
R-DRE-70171	Glycolysis	2.97E-02	2.15E-01	2.13E-02
R-DRE-418594	G alpha (i) signalling events	5.50E-04	1.41E-01	2.23E-02
R-DRE-8964208	Phenylalanine metabolism	2.95E-03	7.06E-03	2.23E-02
R-DRE-70370	Galactose catabolism	1.00E+00	-	2.30E-02
R-DRE-156581	Methylation	5.50E-03	1.00E+00	2.30E-02
R-DRE-428643	Organic anion transporters	2.01E-01	-	2.40E-02
R-DRE-159418	Recycling of bile acids and salts	1.45E-02	-	2.42E-02
R-DRE-193368	Synthesis of bile acids and bile salts via 7alpha...	2.96E-02	-	2.42E-02
R-DRE-70221	Glycogen breakdown (glycogenolysis)	2.41E-03	-	2.49E-02
R-DRE-916853	Degradation of gaba	-	-	2.58E-02
R-DRE-3928662	Ephb-mediated forward signaling	5.30E-01	9.99E-01	2.64E-02
R-DRE-165159	Mtor signalling	7.68E-01	-	2.78E-02
R-DRE-5673001	Raf/map kinase cascade	1.21E-03	1.00E+00	2.97E-02
R-DRE-418555	G alpha (s) signalling events	2.93E-05	1.96E-01	3.01E-02
R-DRE-71403	Citric acid cycle (tca cycle)	5.60E-01	5.98E-02	3.10E-02
R-DRE-389599	Alpha-oxidation of phytanate	1.68E-01	-	4.24E-02
R-DRE-1660662	Glycosphingolipid metabolism	1.70E-02	1.00E+00	4.26E-02
R-DRE-71262	Carnitine synthesis	1.67E-02	-	4.36E-02
R-DRE-5625886	Activated pkn1 stimulates transcription of ar (an...	9.93E-01	1.00E+00	4.75E-02

R-DRE-2299718	Condensation of prophase chromosomes	9.93E-01	1.81E-01	4.75E-02
R-DRE-2559580	Oxidative stress induced senescence	1.48E-02	5.47E-01	4.75E-02

The 57 DE pathways that are linked to DE metabolites in the Zebrafish data. *limma* was applied to assess the significance of DE metabolites, while PLAGE was applied on the metabolite data to prioritise pathway activities in the distal-vs-proximal comparison. For each pathway, a minimum hits of 3 entities was required for PLAGE results to be considered (results with less than 3 hits are represented by a '-' in the table). The Query Builder in GraphOmics was used to filter for DE metabolites that belonged to highly active pathways (based on the metabolite data). A threshold of ≤ 0.05 was used for the p-values. This resulted in 45 compounds across 57 pathways listed above. PLAGE results are shown in the *p-values metabolites* columns and used to sort the table. Additionally the results of running PLAGE on the transcripts and proteins data are also shown in the table under the *p-values transcripts* and *p-values proteins* columns respectively.

Supplementary Table S4: Differentially Expressed Pathways from the Covid-19 Protein Data

Reactome ID	Pathways	p-values proteins	p-values reactome
R-HSA-354192	Integrin signaling	0.00E+00	2.11E-04
R-HSA-8936459	Runx1 regulates genes involved in megakaryocyte d...	0.00E+00	1.89E-03
R-HSA-372708	P130cas linkage to mapk signaling for integrins	0.00E+00	7.69E-06
R-HSA-354194	Grb2:sos provides linkage to mapk signaling for i...	0.00E+00	4.34E-06
R-HSA-114608	Platelet degranulation	0.00E+00	0.00E+00
R-HSA-8964058	Hdl remodeling	0.00E+00	1.64E-04
R-HSA-2022857	Keratan sulfate degradation	0.00E+00	1.43E-01
R-HSA-3656225	Defective chst6 causes mcdc1	0.00E+00	6.25E-02
R-HSA-3656243	Defective st3gal3 causes mct12 and eiee15	0.00E+00	6.25E-02
R-HSA-977225	Amyloid fiber formation	0.00E+00	3.41E-05
R-HSA-166016	Toll like receptor 4 (tlr4) cascade	0.00E+00	9.61E-02
R-HSA-212300	Prc2 methylates histones and dna	0.00E+00	5.79E-02
R-HSA-912446	Meiotic recombination	0.00E+00	9.56E-02
R-HSA-2299718	Condensation of prophase chromosomes	0.00E+00	8.55E-02
R-HSA-5617472	Activation of anterior hox genes in hindbrain dev...	0.00E+00	3.66E-01
R-HSA-427359	Sirt1 negatively regulates rrna expression	0.00E+00	1.50E-02
R-HSA-73728	Rna polymerase i promoter opening	0.00E+00	1.05E-01
R-HSA-5334118	Dna methylation	0.00E+00	3.40E-02
R-HSA-9616222	Transcriptional regulation of granulopoiesis	0.00E+00	1.54E-01
R-HSA-427413	Norc negatively regulates rrna expression	0.00E+00	1.43E-01
R-HSA-5625886	Activated pkn1 stimulates transcription of ar (an...	0.00E+00	1.43E-01
R-HSA-2559582	Senescence-associated secretory phenotype (sasp)	0.00E+00	1.54E-01
R-HSA-73772	Rna polymerase i promoter escape	0.00E+00	1.54E-01
R-HSA-201722	Formation of the beta-catenin:tcf transactivating...	0.00E+00	1.12E-01
R-HSA-1912408	Pre-notch transcription and translation	0.00E+00	1.54E-01
R-HSA-427389	Ercc6 (csb) and ehmt2 (g9a) positively regulate r...	0.00E+00	6.46E-02
R-HSA-5578749	Transcriptional regulation by small rnas	0.00E+00	1.92E-01
R-HSA-9609690	Hcmv early events	0.00E+00	7.64E-01
R-HSA-445989	Tak1 activates nfkb by phosphorylation and activa...	0.00E+00	5.16E-01
R-HSA-933542	Traf6 mediated nf-kb activation	0.00E+00	4.17E-01
R-HSA-879415	Advanced glycosylation endproduct receptor signal...	0.00E+00	2.57E-01
R-HSA-3214815	Hdacs deacetylate histones	4.18E-19	1.54E-01
R-HSA-6802946	Signaling by moderate kinase activity braf mutants	3.85E-18	1.29E-03
R-HSA-5674135	Map2k and mapk activation	3.85E-18	7.50E-04
R-HSA-6802952	Signaling by braf and raf fusions	3.85E-18	5.93E-03
R-HSA-6802948	Signaling by high-kinase activity braf mutants	3.85E-18	4.27E-04
R-HSA-6802955	Paradoxical activation of raf signaling by kinase...	3.85E-18	1.29E-03
R-HSA-9649948	Signaling downstream of ras mutants	3.85E-18	1.29E-03
R-HSA-418594	G alpha (i) signalling events	2.16E-17	2.60E-01
R-HSA-9018519	Estrogen-dependent gene expression	2.88E-17	3.22E-01
R-HSA-917937	Iron uptake and transport	3.00E-10	8.55E-02
R-HSA-1221632	Meiotic synapsis	3.00E-10	3.20E-01
R-HSA-3214847	Hats acetylate histones	5.00E-10	3.36E-01
R-HSA-2559580	Oxidative stress induced senescence	8.00E-10	1.64E-01
R-HSA-5250924	B-wich complex positively regulates rrna expressi...	1.20E-09	1.54E-01
R-HSA-975634	Retinoid metabolism and transport	1.23E-08	1.92E-03
R-HSA-3000178	Ecm proteoglycans	1.59E-08	1.92E-03
R-HSA-8957275	Post-translational protein phosphorylation	2.23E-07	0.00E+00
R-HSA-5694530	Cargo concentration in the er	2.25E-07	7.78E-03
R-HSA-174577	Activation of c3 and c5	4.45E-07	7.80E-09
R-HSA-204005	Copii-mediated vesicle transport	6.07E-07	7.63E-02
R-HSA-6785807	Interleukin-4 and interleukin-13 signaling	9.67E-07	1.54E-01
R-HSA-381426	Regulation of insulin-like growth factor (igf) tr...	3.80E-06	0.00E+00
R-HSA-166665	Terminal pathway of complement	9.60E-06	3.55E-04
R-HSA-8963888	Chylomicron assembly	1.74E-05	2.13E-03

R-HSA-2160916	Hyaluronan uptake and degradation	1.88E-05	1.35E-01
R-HSA-75892	Platelet adhesion to exposed collagen	1.95E-05	3.28E-03
R-HSA-8939236	Runx1 regulates transcription of genes involved i...	2.51E-05	2.82E-02
R-HSA-446353	Cell-extracellular matrix interactions	3.41E-05	1.43E-01
R-HSA-8963901	Chylomicron remodeling	3.84E-05	3.91E-04
R-HSA-3656244	Defective b4galt1 causes b4galt1-cdg (cdg-2d)	4.07E-05	7.78E-03
R-HSA-430116	Gp1b-ix-v activation signalling	9.29E-05	1.36E-03
R-HSA-5686938	Regulation of tlr by endogenous ligand	2.34E-04	1.50E-04
R-HSA-76009	Platelet aggregation (plug formation)	2.48E-04	2.60E-05
R-HSA-3000480	Scavenging by class a receptors	3.45E-04	1.43E-01
R-HSA-6798695	Neutrophil degranulation	8.39E-04	0.00E+00
R-HSA-173736	Alternative complement activation	1.15E-03	2.74E-03
R-HSA-5689880	Ub-specific processing proteases	1.72E-03	3.38E-01
R-HSA-189483	Heme degradation	1.77E-03	8.76E-03
R-HSA-1989781	Ppara activates gene expression	2.37E-03	2.28E-01
R-HSA-2453902	The canonical retinoid cycle in rods (twilight vi...	4.85E-03	1.54E-01
R-HSA-445355	Smooth muscle contraction	6.85E-03	2.32E-01
R-HSA-8964041	Ldl remodeling	7.19E-03	6.00E-02
R-HSA-3000170	Syndecan interactions	8.96E-03	1.90E-02
R-HSA-186797	Signaling by pdgf	1.03E-02	1.54E-01
R-HSA-8963889	Assembly of active lpl and lipc lipase complexes	1.23E-02	8.55E-02
R-HSA-4420097	Vegfa-vegfr2 pathway	1.55E-02	1.10E-01
R-HSA-1236977	Endosomal/vacuolar pathway	1.66E-02	4.51E-01
R-HSA-9033241	Peroxisomal protein import	1.96E-02	7.13E-01
R-HSA-3000471	Scavenging by class b receptors	2.08E-02	1.43E-01
R-HSA-1650814	Collagen biosynthesis and modifying enzymes	2.09E-02	4.13E-01
R-HSA-416476	G alpha (q) signalling events	2.75E-02	2.76E-01
R-HSA-1474228	Degradation of the extracellular matrix	3.31E-02	8.74E-02
R-HSA-202424	Downstream tcr signaling	3.60E-02	2.01E-01
R-HSA-381038	Xbp1(s) activates chaperone genes	4.12E-02	8.30E-01
R-HSA-71336	Pentose phosphate pathway	4.48E-02	1.43E-01

86 DE pathways that are linked to DE proteins in the Covid-19 data. *limma* was applied to assess protein significance, while PLAGE was applied on the protein data to prioritise pathway activities in the severe-vs-healthy comparison. For each pathway, a minimum hits of 3 entities was required for PLAGE results to be considered (results with less than 3 hits are represented by a '-' in the table). From the Query Builder in GraphOmics, significantly changing DE proteins (defined in the original study as having p-values ≤ 0.05 and log fold changes at least ± 0.25), linked to pathways that are also significantly changing (p-values ≤ 0.05) were selected. This resulted in 139 proteins connected to 86 pathways listed above. PLAGE results are shown in the *p-values proteins* columns and used to sort the table. Additionally the results of running Reactome Analysis Service that considers both proteins and metabolites data for over-representation analysis are also shown under the *p-values reactome* column.

Supplementary Figure S5: Differentially Expressed Proteins Linked to Platelet Degranulation Pathway in the Covid-19 Data

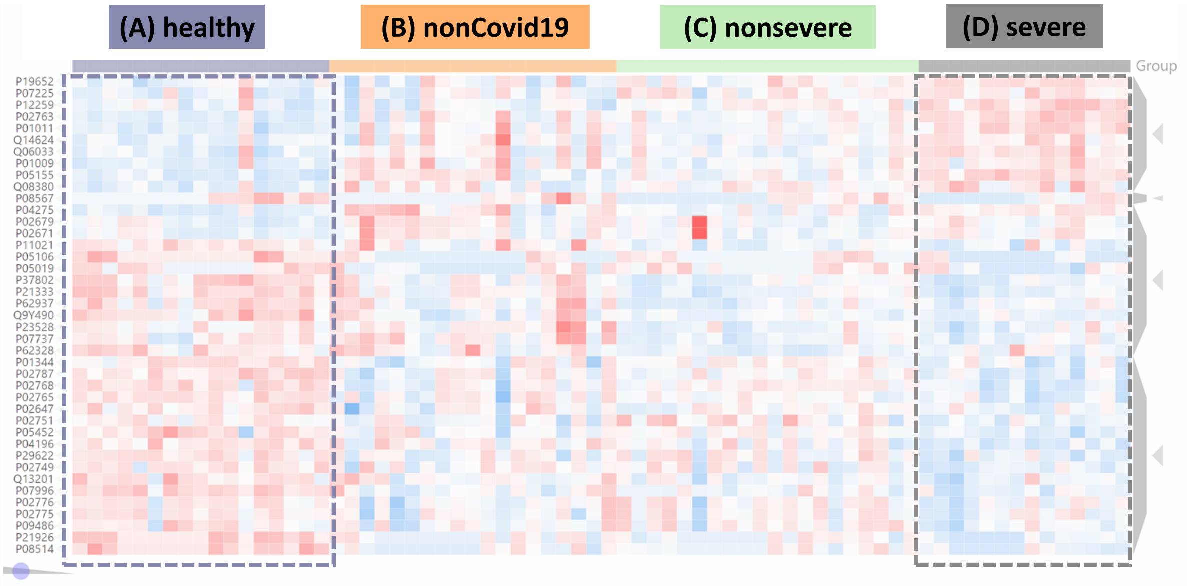

Clustergrammer results for the DE proteins linked to *Platelet Degranulation* pathway. From the Query Builder, significantly changing DE proteins (defined in the original study as having p-values ≤ 0.05 and log fold changes at least ± 0.25), linked to pathways that are also significantly changing (p-values ≤ 0.05) were selected. From this, we filtered for proteins involved in *Platelet Degranulation* pathway from the Data Browser. Two clusters of up-/down-regulated proteins could be observed in the severe-vs-healthy comparison of DE proteins linked to *Platelet Degranulation*.

Supplementary Figure S6: Differentially Expressed Metabolites Linked to Differentially Expressed Pathways in the Covid-19 Data

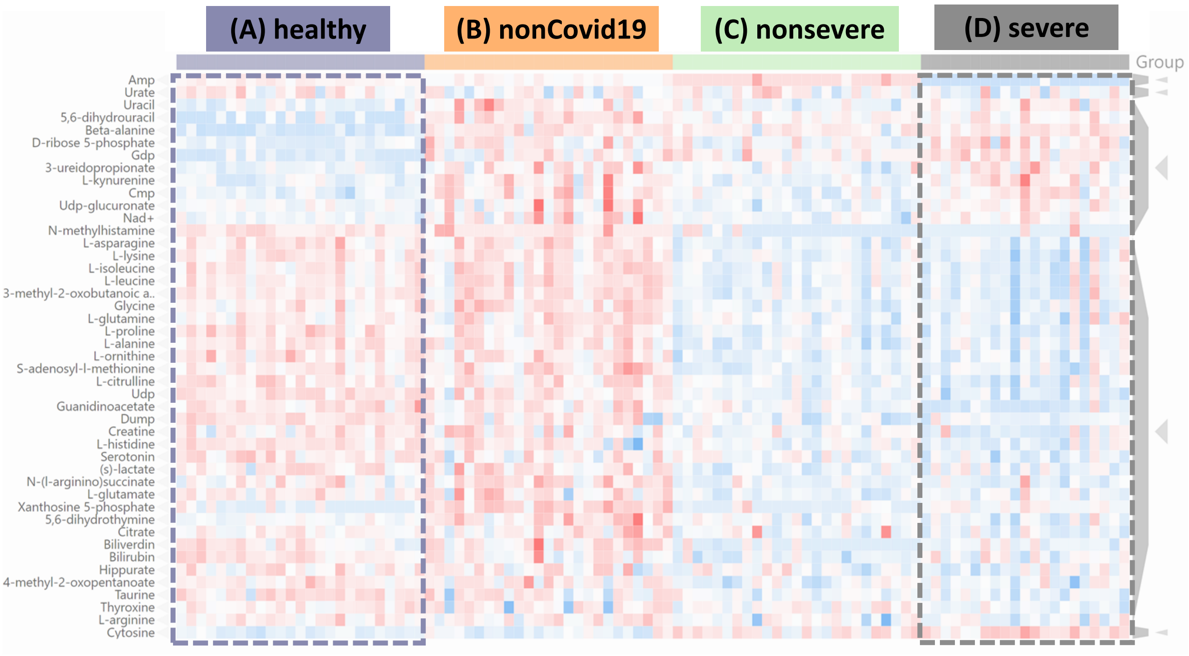

Clustergrammer results for the DE metabolites linked to DE pathways. From the Query Builder, we filtered for DE metabolites linked to DE pathways (p-values ≤ 0.05 for both). Two clusters, one showing an upregulation trend in the severe cohort, and one with downregulation trend could be observed from the Clustergrammer results.
